# *In Vivo* Osseointegration Performance of Titanium Dioxide Coating Modified Polyetheretherketone Using Arc Ion Plating for Spinal Implant Application

**DOI:** 10.1155/2015/328943

**Published:** 2015-10-04

**Authors:** Hsi-Kai Tsou, Meng-Hui Chi, Yi-Wen Hung, Chi-Jen Chung, Ju-Liang He

**Affiliations:** ^1^Department of Materials Science and Engineering, Feng Chia University, No. 100, Wenhwa Road, Taichung City 40724, Taiwan; ^2^Functional Neurosurgery Division, Neurological Institute, Taichung Veterans General Hospital, No. 1650, Taiwan Boulevard Section 4, Taichung City 40705, Taiwan; ^3^Department of Rehabilitation, Jen-Teh Junior College of Medicine, Nursing and Management, No. 79-9, Sha-Luen Hu, Hou-Loung Town, Miaoli County 35664, Taiwan; ^4^Department of Education and Research, Taichung Veterans General Hospital, No. 1650, Taiwan Boulevard Section 4, Taichung City 40705, Taiwan; ^5^Department of Veterinary Medicine, College of Veterinary Medicine, National Chung Hsing University, No. 250, Kuo-kuang Road, Taichung City 40227, Taiwan; ^6^Department of Dental Technology and Materials Science, Central Taiwan University of Science and Technology, No. 666, Buzih Road, Beitun District, Taichung City 40601, Taiwan

## Abstract

Polyetheretherketone (PEEK), which has biomechanical performance similar to that of human cancellous bone, is used widely as a spinal implant material. However, its bioinertness and hydrophobic surface properties result in poor osseointegration. This study applies a novel modification method, arc ion plating (AIP), that produces a highly osteoblast compatible titanium dioxide (TiO_2_) coatings on a PEEK substrate. This PEEK with TiO_2_ coating (TiO_2_/PEEK) was implanted into the femurs of New Zealand white male rabbits to evaluate its *in vivo* performance by the push-out test and histological observation. Analytical results show that AIP can prepare TiO_2_ coatings on bullet-shaped PEEK substrates as implant materials. After prolonged implantation in rabbits, no signs of inflammation existed. Newly regenerated bone formed more prominently with the TiO_2_/PEEK implant by histological observation. The shear strength of the bone/implant interface increases as implantation period increases. Most importantly, bone bonding performance of the TiO_2_/PEEK implant was superior to that of bare PEEK. The rutile-TiO_2_ coatings achieved better osseointegration than the anatase-TiO_2_ coatings. Therefore, AIP-TiO_2_ can serve as a novel surface modification method on PEEK for spinal interbody fusion cages.

## 1. Introduction

The herniated intervertebral disc (HIVD) is the most common spinal disorder. The annulus fibrosus is damaged or weakens when an intervertebral disc is injured such that the nucleus pulposus bulges out or even extrudes posteriorly. This compresses the spinal cord or spinal nerves resulting in pain, paresthesia, muscle atrophy, weakness, and even paralysis, adversely affecting quality of life and ability to work [1, 2]. Severe HIVD frequently requires spinal surgery.

Spondylodesis, or spinal fusion, is the common surgical method. Because the intervertebral disc does not regenerate, a spinal interbody fusion cage is implanted between two vertebrae to support the upper and the lower vertebrae and then fuses after a discectomy. Currently, using a polymeric polyetheretherketone (PEEK) spinal interbody cage is the most common technique. This is a radiolucent to X-rays and noncytotoxic material. In addition, its lower elastic modulus, which resembles that of human cancellous bone, avoids the stress shielding effect and prevents vertebral collapse and osteopenia syndrome [3–5]. Niu et al. [6] indicated that when a titanium (Ti) spinal interbody fusion cage was used, the vertebrae collapsed, enlarging the space between vertebrae and the possibility of cage dislodgement. The stress shielding effect is cited as the cause.

As PEEK is a bioinert [7] and hydrophobic [8] material, its osteoblast attachment and growth are poor. Generally, a few months are needed for osseointegration of the vertebrae into the spinal interbody fusion cage, and patients must wear a neck collar or back brace for several weeks after spondylodesis. To promote the osseointegration of PEEK, two methods, bulk modification and surface modification, have been proposed. The former produces a biomedical composite by mixing PEEK with bioactive hydroxyapatite (HA) [9], tricalcium phosphate (*β*-TCP) [10], and strontium-containing hydroxyapatite (Sr-HA) [11]. The latter treats PEEK with plasma [12] and chemicals [13] and applies a functional coating [14, 15]. However, when PEEK is mixed with bioactive ceramic materials, the tensile strength and toughness of PEEK-based biomedical composites decrease as the amount of materials added increases. Additionally, the elastic modulus of these biomedical composites increases substantially such that the biomechanical property of PEEK is no longer similar to that of human cancellous bone [3–5]. Conversely, surface modification, which only alters the surface of PEEK, does not adversely affect its intrinsic properties. Modifying the surface of PEEK is therefore the better approach when used in a spinal interbody fusion cage.

The surface of titanium dioxide (TiO_2_) generates negatively charged –OH^−^ groups in humid environments, followed by binding with Ca^2+^ and PO_4_^3−^ to form a bone-like apatite, inducing osteoblast attachment and growth [16, 17]. Moreover, TiO_2_ has excellent osseointegration ability based on animal experiments [17, 18]. In a previous report, we successfully deposited a TiO_2_ coating with various ratios of anatase to TiO_2_ (A-TiO_2_) and rutile to TiO_2_ (R-TiO_2_) on a PEEK substrate by low-temperature arc ion plating (AIP) [19]. Their protective [20], photocatalytic activity/antimicrobial properties [21] and osteoblast compatibility [22] were then elucidated comprehensively. These studies demonstrated that TiO_2_ coating significantly improves the osteoblast compatibility of PEEK and in particular R-TiO_2_ phase structure exhibits better performance than A-TiO_2_ phase structure.

This study assesses the* in vivo* osseointegration capacity of PEEK implants coated with TiO_2 _in an animal model. TiO_2_ coatings with A-TiO_2_ or R-TiO_2_ are examined. The aim is to evaluate the ability of the proposed spinal implant in a clinical application to shorten the osseointegration period for the spinal implant and bone tissue.

## 2. Experimental

This research focuses on the methodology used to deposit TiO_2_ coatings with A-TiO_2_ or R-TiO_2_ phase onto a PEEK implant surface under proper deposition parameters. Osseointegration performance is thereafter systematically investigated to determine the effect of the crystal structure on PEEK implants coated with TiO_2_.

### 2.1. Preparation and Characterization of the Implant

**Figure 1:**
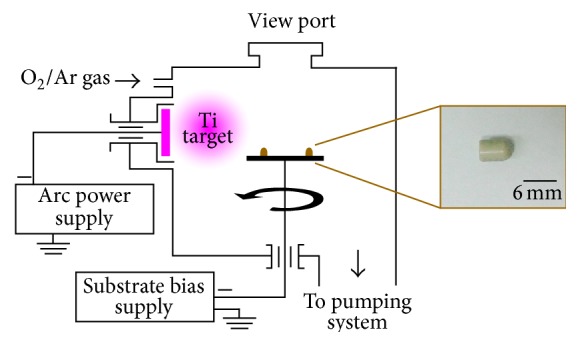
Schematic illustration of AIP equipment and photograph of the bullet-shaped PEEK implant.

The bullet-shaped PEEK implants had a diameter of *φ* 4.0 mm × *L* 6.0 mm. They were cleaned in an ultrasonic alcohol bath and then dried prior to deposition. Deposition was carried out in a typical low-temperature AIP system.  Figure 1 presents the schematic diagram of the AIP equipment and an image of an implant specimen. TiO_2_ coating was prepared in three steps: bombardment with argon ions, deposition of the bottom titanium layer, and deposition of TiO_2_ coating. Bombardment with argon ions cleaned and preheated the substrate, and the bottom titanium layer enhanced adhesion of the substrate to TiO_2_ coating.  Table 1 shows detailed deposition conditions, through which TiO_2_ coatings with the full crystal structure of A-TiO_2_ and R-TiO_2_ were obtained by controlling target current and substrate bias voltage.

The crystal structures of TiO_2_-coated PEEK implants were analyzed using a Bruker D8 multipurpose thin-film X-ray diffractometer with Cu K*α* radiation (1.540 Å). A Hitachi S-4800 cold field emission scanning electron microscope (FESEM) was used to observe the surface and cross-sectional morphologies of TiO_2_-coated PEEK implants.

### 2.2. Surgical Procedure

The animal experiment protocol was reviewed and approved by the Institutional Animal Care and Use Committee (IACUC) of Taichung Veterans General Hospital. Twenty-four specific pathogen-free (SPF) New Zealand white male rabbits were divided randomly into three groups,* that is*, eight rabbits per group. The PEEK implant, A-TiO_2_/PEEK implant or R-TiO_2_/PEEK implant, was implanted into a distal femur in each rabbit.

**Figure 2:**
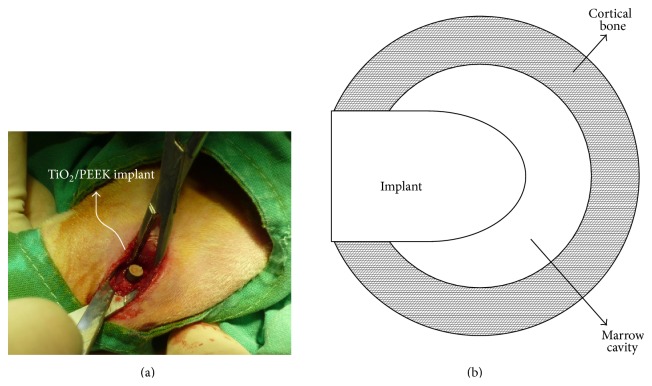
(a) Intraoperative image of implant placement. (b) Illustration of implant placement.

All surgical devices, instruments, and specimens were sterilized to prevent bacterial infections. The rabbits were anesthetized by intravenous injection of anesthetics and antibiotics. A scalpel was utilized to incise the skin and muscle of the leg and expose the femur surface. Implantation sites were prepared using an orthopedic drill with a diameter of 3 mm. The holes were widened gradually until the final size was suitable for the 4-mm implant. During drilling, the area was continuously flushed with saline to reduce mechanical and thermal damage to the femur. The implant was then inserted into the hole and pushed into the marrow cavity using finger pressure (Figure 2). The fascia and skin were then sutured with 3-O Nylon suture. One implant was inserted into left and right femora of each rabbit. Betadine was again used to disinfect the surgical area. After surgery, antibiotics were administered to prevent wound infection. Wound healing was monitored continuously.

After implantation periods of 4, 8, and 12 weeks for groups PEEK, A-TiO_2_/PEEK, and R-TiO_2_/PEEK, respectively, two rabbits in each time point for each group were euthanized with carbon dioxide and their femur with the implant was excised. The femora samples were then placed into formaldehyde (37%) to fix bone tissues. Subsequently, the three femora samples were used to evaluate the fixation degree of implant/bone tissues by push-out test, and the other one is used to examine the bone/implant interface by histological observation. Finally, the total eighteen rabbits were used in the animal experiment, and the remaining six rabbits were provided against unexpected needs.

### 2.3. Push-Out Test

The excised femora sample was mounted onto a special platform using epoxy. An Algol JSV-H1000 automatic vertical test stand with a Handy HF-1000 digital force gauge was used to conduct the push-out test under a displacement speed of 1 mm/min. Peak force between bone tissues and the implant was acquired from the load-displacement curves. The thickness of cortical bone contacting the implant was measured and calculated as the mean of measurements at five sites chosen randomly for determining the bone-implant contact area. Each piece of data of the push-out test was calculated from three femora samples to give an average result and a standard deviation. In addition, those data were also analyzed by *t*-test for statistical significance, and *p* values < 0.05 were considered significant. The shear strength between bone tissues and the implant was derived as follows:

(1) Shear  strength  (MPa)=Peak  forceBone-implant  contact  area=Peak  forceπ×Implant  diameter×Cortical  bone  thicknessN/mm2

After the push-out test, the disrupted implants were fixed in formaldehyde solution and then dehydrated in ethanol solutions graded at 75–100%. To further assess osseointegration, the fracture microstructures between the implant surface and bone tissues were examined by FESEM with energy dispersive spectrometer (EDS) element mapping for failure mode analyses.

### 2.4. Histological Observation

To examine the interface between bone and the implant, the excised femora were dehydrated in graded ethanol solution, followed by cold mounting in epoxy using a Struers CitoVac vacuum impregnation unit, allowing the epoxy to penetrate bone tissues. A Struers Accutom-50 precision cut-off and grinding machine was utilized to slice off 100-*μ*m thin sheets. Specimens were then stained with Hematoxylin and Eosin (H&E) and their histomorphology was characterized* via* optical microscopy (OM) to assess the bone bond condition.

## 3. Results

### 3.1. Crystal Structure and Microstructure of TiO_2_-Coated Implants

**Figure 3:**
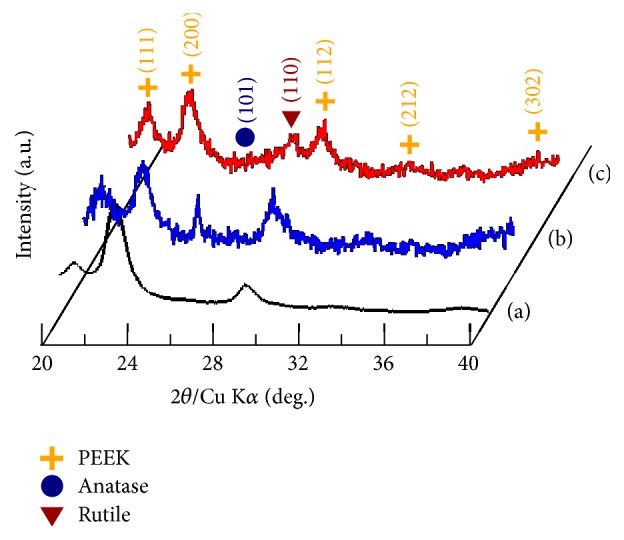
XRD patterns of the (a) PEEK implant, (b) A-TiO_2_/PEEK implant, and (c) R-TiO_2_/PEEK implant.

Figure 3 shows the X-ray diffraction (XRD) patterns of the three implants. A TiO_2_ coating with complete crystal structures was deposited successfully on the irregular PEEK implant surface using low-temperature AIP (<170°C). No XRD peaks corresponding to the PEEK implant before and after TiO_2_ coating process changed, suggesting that the PEEK substrate was not degraded during surface modification with TiO_2_ coating. The phase structure of TiO_2_ coating, by the XRD pattern, can be controlled by adjusting the target current and substrate bias voltage during deposition* via* the AIP system. The growth mechanism behind this has been previously investigated [21, 23].

**Figure 4:**
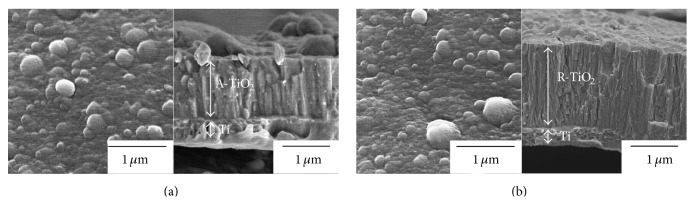
FESEM surface and cross-sectional morphologies of the (a) A-TiO_2_/PEEK implant and (b) R-TiO_2_/PEEK implant.

Figure 4 shows the FESEM surface and cross-sectional morphologies of the TiO_2_-coated PEEK implants. Due to the high ionization efficiency and high ion kinetic energy in the AIP process [19], TiO_2_ coating was continually bombarded with titanium ions during the growth process, increasing substrate temperature and adatom mobility. Thus, TiO_2_ coating appears as dense crystalline columnar structures. The film thickness of A-TiO_2_ and R-TiO_2_ coatings was 1.31 ± 0.06 *μ*m and 1.61 ± 0.04 *μ*m, respectively (Figures 4(a) and 4(b)). The reasons for the effect of coating parameters on TiO_2_ coating thickness were elucidated previously [21]. Based on the surface morphology for both A-TiO_2_ and R-TiO_2_ coatings, macroparticles, which have been known to be a result of metal titanium microdroplets in conjunction with titanium ions emitted from the titanium cathode during the deposition process in AIP system, were observed in Figure 4 [22]. These microdroplets react with oxygen during their flight to the substrate surface to form partial or full oxide, called as “macroparticles,” thereby increasing surface roughness of the TiO_2_-coated implants. Based on the previous results, the average roughness of A-TiO_2_ and R-TiO_2_ coatings is 1.49 ± 0.08 *μ*m and 1.58 ± 0.06 *μ*m, respectively, using a surface roughness tester [22]. Fortunately, a roughened surface promotes osteoblast cell proliferation and cell differentiation due to the induced release of growth factors and cytokines from the adhered osteoblast cells [24, 25]. In addition, roughened surfaces promote mechanical interlocking between bone tissues and implant [26]. According to the explanation of this phenomenon, subsequent* in vivo* osseointegration performance will provide a positive benefit.

### 3.2. Clinical Observations

During the experimental period, two rabbits died of diarrhea with suspected foodborne* E. coli* at 8 weeks after implantation (confirmed by autopsy). The remaining rabbits did not present any signs of inflammation or an adverse tissue response, confirming that the PEEK implant and TiO_2_ coating are not cytotoxic.

### 3.3. Shear Strength of the Bone-Implant Interface

**Figure 5:**
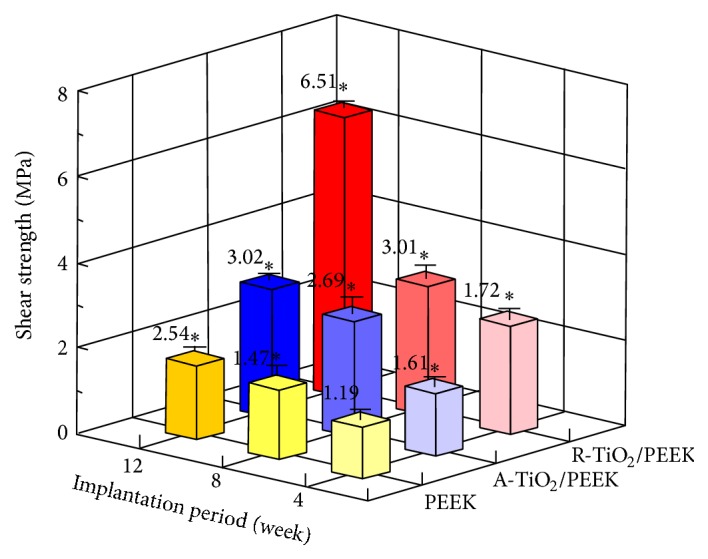
Shear strength between bone tissues and implant for the three implants at 4, 8, and 12 weeks after implantation. ^*∗*^ *p* < 0.05 compared to PEEK implant at 4 weeks after implantation.

Successful osseointegration is characterized by stability between implant and bone tissues [27]. The push-out test can precisely quantify the degree of fixation between an implant and bone tissues [28].  Figure 5 shows push-out test results for the three implants at 4, 8, and 12 weeks after implantation. The shear strength between bone tissues and implant increased as implantation time increased. At 12 weeks, the shear strength of the PEEK implant was 2.54 MPa, that of the A-TiO_2_/PEEK implant was 3.02 MPa, and that of the R-TiO_2_/PEEK implant was 6.51 MPa, leading to the conclusion that the PEEK implant had the poorest shear strength. Shear strength can be enhanced by TiO_2_ coating when the TiO_2_-coated PEEK specimen is implanted in bone tissues. The R-TiO_2_ coating had the best fixation.

**Figure 6:**
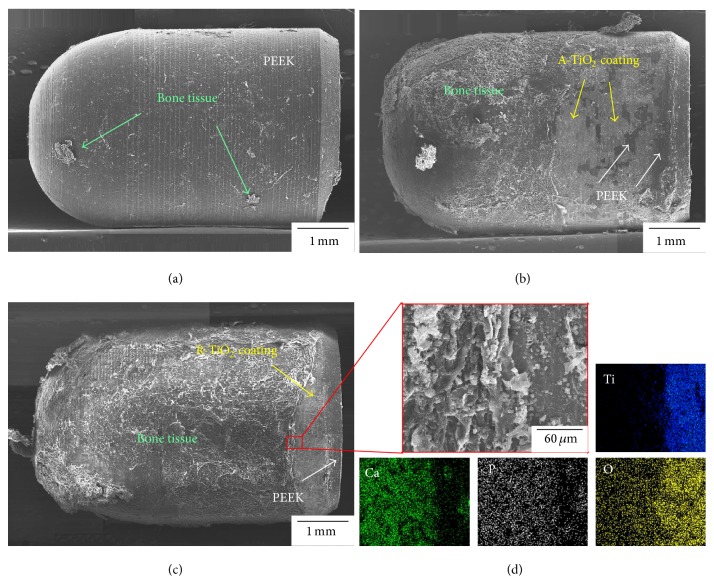
Fracture morphology of (a) PEEK implant, (b) A-TiO_2_/PEEK implant, and (c) R-TiO_2_/PEEK implant with (d) its composition analysis of bone tissues and implant interface after the push-out test at 12 weeks of implantation.

To identify the failure mode between implant and bone tissues after the push-out test, FESEM was applied to observe fracture morphology of the implant surface at 12 weeks after implantation as shown in Figure 6. New bone had fully peeled off from the surface of the unmodified PEEK implant (Figure 6(a)), indicating that failure occurred at the bone/PEEK implant interface. Thus, the osseointegration capacity of an unmodified PEEK implant is poor. After TiO_2_ coating was applied, the large area of residual new bone tissues adhered to the surface of the two TiO_2_/PEEK implants (Figures 6(b) and 6(c)), where evident composition analysis of residual bone tissue on the R-TiO_2_/PEEK implant was confirmed by EDS element mapping in Figure 6(d). These analytical results indicate that TiO_2_ coating has superior ability to induce new bone growth and achieve bone ingrowth. However, slight detachment of A-TiO_2_ coating was also found in the A-TiO_2_/PEEK implant. It results in failure of the A-TiO_2_/PEEK implant which occurred at internal fracture of bone tissues and interface failure of A-TiO_2_ coating/PEEK implant interface. The R-TiO_2_/PEEK implant surface was almost completely covered with new bone tissues and the R-TiO_2_ coating at the end of the R-TiO_2_/PEEK implant did not detach from PEEK implant surface. Therefore, failure mode for the R-TiO_2_/PEEK implant was internal fracture of bone tissues. Overall, the R-TiO_2_/PEEK implant exhibits good film adhesion between the R-TiO_2_ coating and PEEK implant as well as good bonding between new bone tissue and the R-TiO_2_/PEEK implant, implying excellent osseointegration at the implant/bone interface.

### 3.4. Bone Bonding Response at the Bone-Implant Interface

**Figure 7:**
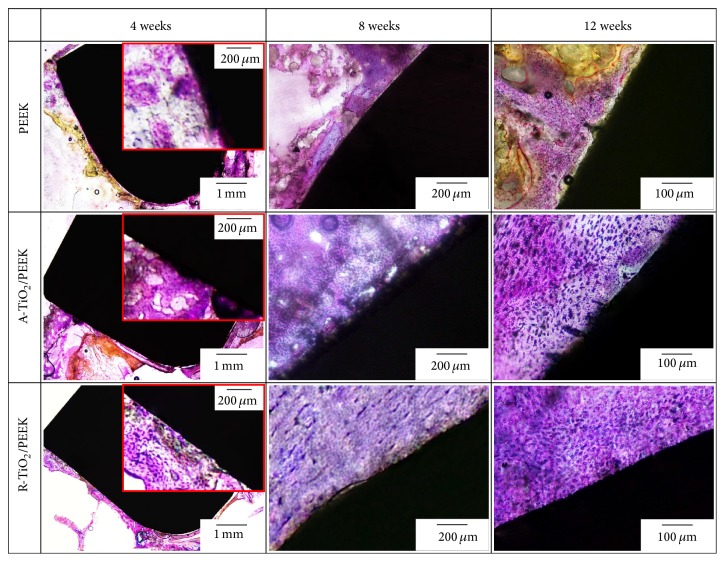
Histological sections of the PEEK and TiO_2_/PEEK implants at 4, 8, and 12 weeks after implantation.

Osseointegration is defined as direct anchorage of an implant by the formation of bony tissue around the implant without fibrous tissue at the bone-implant interface [29]. The effects of an implant on new bone growth can be determined by histological observation.  Figure 7 shows histological sections of the three implants at 4, 8, and 12 weeks after implantation. At 4 weeks, new bone generated by bone remodeling had formed mature lamellar bone that directly contacted the TiO_2_/PEEK implant, indicative of excellent osseointegration performance. Thus, the TiO_2_ coating exhibits good osteoblast compatibility and rapidly activates bone remodeling. Subsequently, the coating induced adhesion and proliferation of osteoblasts to the implant surface and differentiation into osteocytes for the production of new bone tissues and later bone bonding. Conversely, new lamellar bone on the surface of the unmodified PEEK implant was not completely mature and did not bond fully with the implant. The response of the implant in the marrow cavity (located far from the cortical bone) at 4 weeks after the implantation indicated that regenerated bone tissues were growing onto the TiO_2_/PEEK implant surface. This new bone is the result of bone tissue repair, which proliferated from the endosteum of cortical bone. Due to the osteoconductive effect, new bone tissues grew inward to the implant surface in the marrow [30]. These findings indicate that the TiO_2_ coating has excellent osteoconductivity and promotes new bone growth on the TiO_2_/PEEK implant surface with connections to cortical bone. However, the surface of the unmodified PEEK implant was covered with fibrous tissue, implying that bone bonding did not occur between the implant and cortical bone. Fibrous tissue growth is likely caused by the micromovement in the PEEK implant and poor stability in the early implantation period [31].

When the implant period was extended to 8 weeks, immature osteogenesis existed in the cortical bone around the unmodified PEEK implant. New bone was maturing at 12 weeks after implantation; however, fibrous tissue was identified at the interface between the unmodified PEEK implant and bone tissues. This indicates that the osseointegration capacity of the unmodified PEEK implant was very limited, even when the implantation period was extended. At 8 weeks after implantation, histological sections of the TiO_2_/PEEK implants in the marrow cavity reveal that new bone was maturing and osteocytes covered the TiO_2_/PEEK implant surface, showing that the TiO_2_ coating, due to its osteoconductive effect, can trigger quick bone remodeling. The new bone was mature and closely integrated with the TiO_2_ coating in the cavity at 12 weeks after implantation (Figure 7). However, by comparison, for TiO_2_ coatings with different phase structures, the degree of bone bonding between new bone and the R-TiO_2_/PEEK implant was significantly better than that between A-TiO_2_ and the PEEK implant. In addition, some gaps existed between the A-TiO_2_ coating and new bone in some areas and detachment of the A-TiO_2_ coating was found.

## 4. Discussion

Reports indicate that the success rate of implantation is determined mainly by osseointegration [32]. Osseointegration is measured as the stability between an implant and bone tissues. Implant stability can then be divided into primary stability (just implanted) and secondary stability. Primary stability is due to mechanical engagement with cortical bone, and it is affected by the quality of bone into which the implant is inserted, the surgical procedure, and implant type. Secondary stability is regeneration and remodeling of bone tissues around the implant after insertion, that is, osseointegration [33]. To achieve stability between bone and implant, one must increase the osseointegration rate by roughening the implant surface or creating bioactivity on the surface of the implant. The relevant literatures reported that roughening an implant surface by only 1 *μ*m can increase contact bone growth [34]. A rough and porous surface can increase mechanical interlocking of an implant with bone tissue and induces adhered osteoblasts to secrete growth factors and cytokinins which subsequently increase the proliferation, differentiation, and fusion capacity of osteoblasts [26]. On the other hand, when TiO_2_ is immersed in simulated body fluid (SBF), its surface binds with water molecules and forms negatively charged –OH^−^ functional group. This negatively charged functional group absorbs Ca^2+^ to the TiO_2_ surface for nucleation and attracts PO_4_^3−^ and Ca^2+^ to form apatite layer onto the TiO_2_ surface [16, 35]. Additionally, Ca^2+^ on the TiO_2_ surface can also absorb protein [36]. These changes in the TiO_2_ surface will induce osteoblasts to attach and grow, increasing bone tissue growth and thus implant stability [37].

Notably, PEEK is a bioinert [7] and hydrophobic [8] material that does not induce osteoblasts to attach and grow. Immature bone tissue did not attach well to the surface of the PEEK implant and unfavorable fibrous tissue was produced, indicating poor stability and poor bonding with bone tissues (Figure 7). The push-out test results indicate that the bone tissues detached completely from the PEEK implant surface, resulting in unsatisfactory shear strength between implant and bone tissues. On the other hand, the average surface roughness of the TiO_2_ coating formed by AIP was about 1.5 *μ*m reported in a previous study [22]. Researchers believe that the rough surface initially enhances the mechanical interlocking of the TiO_2_/PEEK implant with bone tissues, thus improving primary stability. Furthermore, the TiO_2_ coating surface has negatively charged –OH^−^ functional groups. These groups produce a hydrophilic surface and induce Ca^2+^ and PO_4_^3−^ nucleation, followed by the formation of apatite layer. The reaction provides a good growth environment of osteocytes to trigger quickly bone remodeling. Therefore, the secondary stability of the TiO_2_/PEEK implants is enhanced by the osteoconductive effect, which induced cortical bone endosteum to regenerate bone tissue and grow inward into the marrow covering the TiO_2_/PEEK implant. Finally, mature regenerated bone tissue bonded with the TiO_2_/PEEK implant, indicating superior osseointegration.

Furthermore, the effect of TiO_2_ phase structure on osteoblast compatibility is demonstrated in our previous study [22]. The analytical results reveal that the AIP-TiO_2_-coated specimens (including different ratios of A-TiO_2_ and R-TiO_2_ phase structures) had better osteoblast cell adhesion, proliferation, differentiation, and bone formation (osteopontin, osteocalcin, and calcium content) than bare PEEK polymers. The R-TiO_2_ coating particularly possesses best osteoblast compatibility due to the abundance of negatively charged –OH^−^ groups on its surface. This finding agrees with push-out test results (Figure 5) and histological examination findings (Figure 7) in this study, indicating that shear strength and bone bonding response of the R-TiO_2_ coating were significantly better than those of the A-TiO_2_ coating. Our previous work also reported that film adhesion and protection properties of the A-TiO_2_ coating are slightly worse than those of the R-TiO_2_ coating [20]. Hence, although the A-TiO_2_ coating provides osteoconductivity for bonding with bone tissues (lower than that of R-TiO_2_), the A-TiO_2_ coatings unfortunately detached during the long implantation period, leading to limited improvement in shear strength from bone tissue such that the shear strength of the A-TiO_2_/PEEK implant was only 1.19 times greater than that of the PEEK implant and that of the R-TiO_2_/PEEK implant was 2.6 times greater.

Overall, the AIP-TiO_2_ coating surface modification technique improves the osseointegration of PEEK implants. In terms of bone bonding between implants and new bone tissues, performance from best to worst is R-TiO_2_/PEEK implant > A-TiO_2_/PEEK implant ≫ PEEK implant. However, overall strength of the new bone/coating adhesion and coating/substrate adhesion follows the order R-TiO_2_/PEEK implant ≫ A-TiO_2_/PEEK implant > PEEK implant.

## 5. Conclusions

This study applied AIP to prepare A-TiO_2_ and R-TiO_2_ coatings on bioinert PEEK implants. The improvement of osseointegration capacity in PEEK implant after AIP-TiO_2_ coating surface modification was systemically investigated. Analytical results indicate that surface roughness and surface electrochemical properties of the TiO_2_ coating can improve the mechanical interlocking and osteoinductive and osteogenic activity of the TiO_2_/PEEK implant, further enhancing stability between implant and bone tissues. Therefore, the degree of bone bonding response and shear strength at the interface between the TiO_2_/PEEK implants and regenerated bone tissues are significantly better than those of the bare PEEK implant. The R-TiO_2_/PEEK implant achieves better osseointegration than the A-TiO_2_/PEEK implant due to the abundance of negatively charged –OH^−^ groups on its surface. Further study of the R-TiO_2_/PEEK implant for clinical application as a spinal implant is warranted.

## Figures and Tables

**Table 1:** Parameters for deposition of TiO_2_ coatings.

Deposition parameter	60 A 0 V	90 A 50 V
Target	Ti	
Working pressure (Pa)	0.5	
Deposition time (min)	20	
Target voltage (V)	20	
Target current (A)	60	90
Substrate bias (−V)	0	50
Crystal structure of TiO_2_ coating	A-TiO_2_	R-TiO_2_
